# Novel Cuproptosis-Related Gene Signature for Precise Identification of High-Risk Populations in Low-Grade Gliomas

**DOI:** 10.1155/2023/6232620

**Published:** 2023-02-13

**Authors:** Ping Chen, Hailing Han, Xuejie Wang, Bing Wang, Zhanfeng Wang

**Affiliations:** ^1^Department of Neurosurgery, The Second Affiliated Hospital of Hengyang Medical College, Hengyang, 421000 Hunan, China; ^2^Department of Neurosurgery, China-Japan Union Hospital of Jilin University, Changchun, 130000 Jilin, China; ^3^Department of Radiation Oncology, The Second Hospital of Jilin University, Changchun 130022, China

## Abstract

**Background:**

Patients with low-grade glioma (LGG) have wildly varying average lifespans. However, no effective way exists for identifying LGG patients at high risk. Cuproptosis is a recently described form of cell death associated with the abnormal aggregation of lipid acylated proteins. Few investigations have been conducted on cuproptosis-associated genes and LGG thus far. The purpose of this research is to establish a predictive model for cuproptosis-related genes in order to recognise LGG populations at high risk.

**Methods:**

We analyzed 926 LGGs from 2 public datasets, all of which were RNA sequencing datasets. On the basis of immune scores, the LGG population was split into different risk categories with X-tile. LASSO and Cox regressions were employed to filter cuproptosis-associated genes and construct prediction models. The accuracy of the predictive models was measured by using TCGA internal validation set and the CGGA external validation set. In addition, LGG immune cell infiltration was viewed using CIBERSORT and ssGSEA algorithms and correlation analysis was done with cuproptosis-related genes. Finally, immune escape capacity in LGG low- and high-risk groups was evaluated using the TIDE method.

**Results:**

The prediction model constructed by four cuproptosis-related genes was used to identify high-risk populations in LGG. It performed well in training and all validation sets (AUC values: 0.915, 0.894, and 0.774). Meanwhile, we found that FDX1 and ATP7A in the four cuproptosis-related genes were positively correlated with immune response, while GCSH and ATP7B were opposite. In addition, the high immune score group had a lower TIDE score, indicating that their immune escape capacity was weak.

**Conclusion:**

High-risk individuals in LGG can be reliably identified by the model based on cuproptosis-related genes. Furthermore, cuproptosis is closely related to tumor immune microenvironment, which gives a novel approach to treating LGG.

## 1. Introduction

As an important subtype of glioma, LGG accounts approximately for 20% of intracranial tumors. Currently, the median survival time of LGGs is between 5.6 and 13.3 years [1]. LGG appears to have a good prognosis compared to the shorter survival of glioblastoma. However, it has a high potential to evolve into malignant tumor, and seventy percent or more of LGGs can mutate into secondary glioblastoma or anaplastic gliomas [2]. Thus, given the high incidence and high malignant transformation rate of LGG, the search for reliable prognostic indicators is of the utmost importance.

The 2016 revision of the WHO classification includes a number of biomarkers—IDH-1, TP53, MGMT, and 1p/19q codeletion—that are important in determining the outcome of LGG patients [3]. However, these markers are expressed in specific pathological types of LGG and do not allow for a correct and comprehensive risk stratification of LGG. As a result, there is a pressing need to create efficient models for pinpointing high-risk LGG groups.

The immune microenvironment is closely related to tumor progression and has been used to predict cancer prognosis [4]. It was found that low level of infiltrating immune cells might be an element in the better prognosis of LGG than glioblastoma. The LGG prognosis was highly variable based on the level of immune cell infiltration, showing that the prognosis of glioma was closely related to immunity [5, 6]. In order to more visually reflect immune cell infiltration within the tumor, a calculation method called immune score has been introduced [7]. Recently, a new cell death pathway called cuproptosis has come into people's view. Copper binding to lipid acylated components of the tricarboxylic acid cycle leads to proteotoxic stress and, eventually, cell death, a process known as cuproptosis [8]. Genes involved in cuproptosis were found to have a strong correlation with the degree of immune infiltration in patients with clear cell renal cell carcinoma, according to research by Bian et al. [9]. As of yet, cuproptosis and LGG immune infiltration have not been studied. It is also important to investigate whether this novel cell death pattern influences LGG prognosis. This finding holds promise as a potential new biomarker for assessing LGG patients' prognoses.

In this paper, we first used cuproptosis-related genes to construct a model to identify immune high-risk LGG populations and to explore the relevance and possible mechanisms of cuproptosis-related genes to immune regulation. Here, we used immune score as a filter to identify high-risk people in The Cancer Genome Atlas (TCGA) LGG cohort. A model was then developed to predict this high-risk group of patients based on cuproptosis-related genes using the LASSO regression. In addition, we evaluated the accuracy of the model's predictions against both TCGA and the China Glioma Genome Atlas (CGGA) databases. Subsequently, we evaluated the variations in immune cell infiltration across groups with varying immune score using the CIBERSORT and ssGSEA algorithms and further explored the relevance of cuproptosis-related genes to these immune cells and immune function. Finally, the TIDE method was utilised to predict the possible efficacy of immunotherapy in this subset of high-risk LGG sufferers.

## 2. Materials and Methods

### 2.1. LGG Datasets

RNA-seq data and clinical information of LGG were obtained from TCGA (https://portal.gdc.cancer.gov/) and CGGA (http://cgga.org.cn/) databases, respectively. LGG patients with information on survival status, survival time, pathology, age, gender, and tumor grade were brought into this study. The gene expression results obtained were fragments per kilobase transcript per million mapped reads (FPKM), which were subsequently transformed into subsequent transcripts per million (TPM). In particular, to validate the external applicability of the model, we did not debatch the two datasets. Each LGG participant's immune scores were calculated using the ESTIMATE methodology [7].

### 2.2. Identify High-Risk Groups

The immune score was combined with other clinical information of the patients in a multivariate Cox regression analysis to identify whether LGG patients' immune scores were an independent predictive factor for prognosis. Among LGG patients in TCGA, we used X-tile to obtain cutoff values according to their immune scores and segregated them into those with high immune scores and those with low ones [10]. Subsequently, survival curves were constructed using the Kaplan–Meier method to compare whether survival rates varied across LGG subgroups based on immunological score. The same method was used to process data from LGG patients included in this research in GCGA.

### 2.3. Predictive Model Building and Evaluation

Cuproptosis-related genes were obtained through Tsvetkov et al.'s study [8]. To begin with, we analyzed whether cuproptosis-related genes' expression differed between immune score groups. By univariate Cox analysis, the cuproptosis-related gene strongly linked to LGG patient survival was screened according to the *p* value less than 0.05. Based on survival-related cuproptosis-related genes and different immune score groups, the R package “glmnet” was used to run LASSO regression analysis to further choose the most relevant markers for distinguishing distinct immunological score groups. In order to stabilise the results as much as possible, we used 5-fold cross-validation and determined the optimal penalty parameter *λ* based on 1 − SE (standard error). At the same time, we calculated the risk score based on the coefficients of cuproptosis-related genes included in the model and their expression levels. With the “survivalROC” R package, we adopt the receiver operator characteristic curve (ROC) and the area under the curve (AUC) for the predictive model's internal and external validation.

### 2.4. Immune Cell Infiltration and Analysis

Using the CIBERSORT approach, we mapped the distribution of 22 immune cells throughout LGG tissues [11]. Cases with *p* values less than 0.05 were retained for subsequent differential analysis of immune cell infiltration in different immune score groups. In addition, the acquired significantly differently expressed immune cells were then sorted into high and low expression groups based on the median value of their expression levels, and the KM curve was used to analyze their impact on the prognosis of LGG patients. Finally, we used the “corrplot” package for immune cell correlation analysis.

### 2.5. Correlation Analysis of Cuproptosis-Related Genes with Immune Cells and Immune Function

Based on previous literature, a gene set containing 29 immune signatures was found [12]. The LGG cohort of TCGA was then subjected to a single-sample gene set enrichment analysis (ssGSEA) to see how enriched the 29 immune signatures were in each LGG sample [13, 14]. Finally, the “ggcorrplot” package was used to analyze the association of cuproptosis-related genes with immune cells and immune function, and a heat map was made to show the results.

### 2.6. Evaluation of Immune Escapes

Immune escape is a challenge for immunotherapy. A tumor's ability to evade the immune system is measured using a scale called the Tumor Immune Dysfunction and Exclusion (TIDE) score. The TIDE algorithm, on the basis of simulating two mechanisms of tumor immune evasion: T cell dysfunction induction in cancers with high cytotoxic T lymphocyte (CTL) infiltration and T cell infiltration prevention in tumors with low CTL levels, is used to assess the potential for tumor immune evasion [15]. TIDE scores were performed on different immune score groups to assess their immune escape ability.

### 2.7. Statistical Analysis

All data analysis in this study was implemented based on the R software (version 4.1.1), except for the use of X-tile (3.6.1) software to obtain optimal immune score cutoff values. Descriptive analyses of baseline characteristics of TCGA and CGGA datasets were performed, with continuous variables shown as mean ± standard deviation and categorical variables shown as frequencies. Univariate and multivariate COX regression analyses were used to screen for prognosis-related risk factors. LASSO regression is used to screen cuproptosis-related genes further and build prediction models. The ROC curves were used to evaluate the model's predictive accuracy. The OS of LGG patients was compared across groups with various immunological scores using the Kaplan–Meier method with a log-rank test. To evaluate the degree of association, we turned to Spearman's correlation analysis. *p* values for significant differences were set at less than 0.05.

## 3. Results

### 3.1. Patients' Characteristics

The study comprised 926 LGG patients, 506 from TCGA database and 420 from the CGGA database. Immune score threshold was 303.4, and patients were then separated into categories with high and low immune scores (X-tile plots are shown in Supplementary Figure 1). There were 782 patients in the group with a low immune score, and 248 of them passed away over a median of 31.2 months of follow-up. 144 LGG patients were assigned to the high immune score group, and 75 patients died during a median of 26.6 months of follow-up. Additional clinical information of patients in both databases is shown in Table 1.

### 3.2. Multivariate Cox Analysis for OS

**Figure 1:**
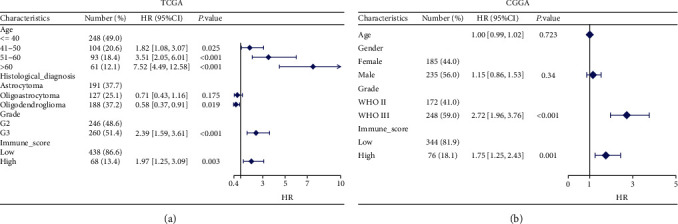
Results of multivariate Cox regression analysis on the OS of (a) LGG cohort in TCGA and (b) LGG cohort in CGGA.

**Figure 2:**
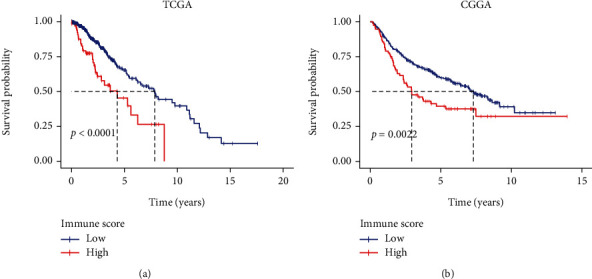
Kaplan–Meier curves for OS of patients in the high and low immune score groups in (a) TCGA and (b) CGGA.

Figure 1 shows the results of the multivariate Cox analysis. It was clear that grade and immune score were LGG patients' independent prognostic factors in both datasets, and high immune score was a prognostic risk factor (hazard ratios (HR): 1.86, 95% confidence interval (CI), 1.18-2.94, *p* = 0.008; hazard ratios (HR): 1.75, 95% confidence interval (CI), 1.25-2.43, *p* = 0.001). And the median survival for LGG patients with a high immune score was dramatically lower than that of those with a low immune score (Figure 2).

### 3.3. Construction of Predictive Model from TCGA

**Figure 3:**
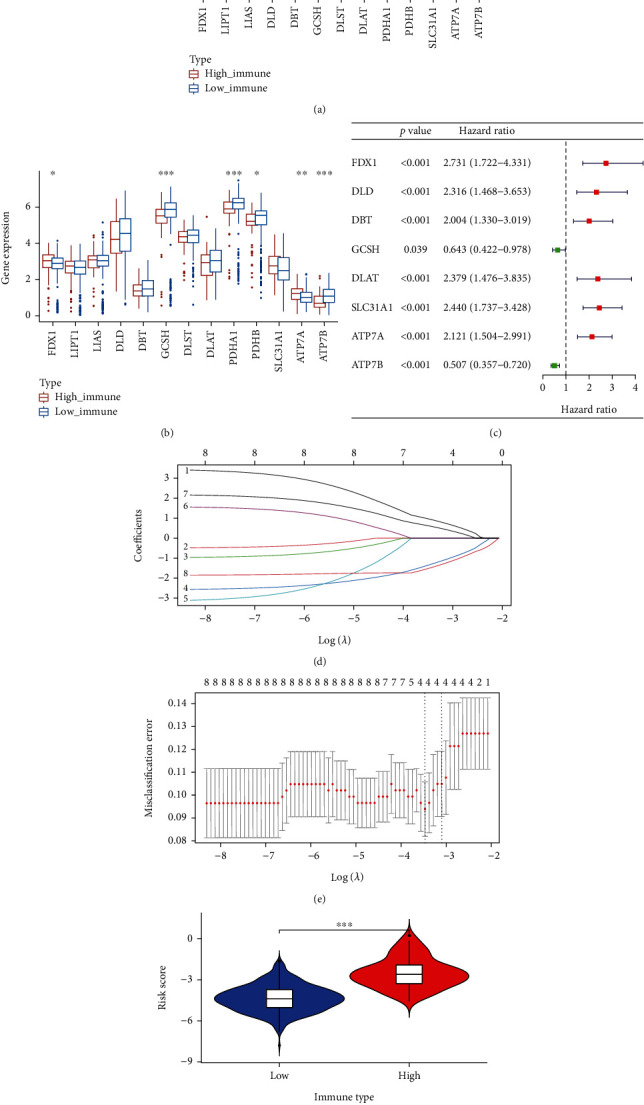
Cuproptosis-related gene screening and prediction model construction by LASSO regression. (a) Cuproptosis-related gene expression in the high and low immune score groups in TCGA cohort. (b) Cuproptosis-related gene expression in the high and low immune score groups in the CGGA cohort. (c) Forest plot of hazard ratios demonstrating the prognostic values of cuproptosis-related genes. (d) LASSO coefficient profiles of the 8 genes in LGG. (e) A coefficient profile plot was generated against the log (lambda) sequence. Selection of the optimal parameter (lambda) in the LASSO model for LGG. (f) Association between immune score and risk score. Adjusted *p* values were showed as ^∗^*p* < 0.05, ^∗∗^*p* < 0.01, and ^∗∗∗^*p* < 0.001.

In TCGA and CGGA datasets, most cuproptosis-related genes showed substantial expression differences between immunological groups (Figures 3(a) and 3(b)). Using univariate Cox regression, we identified eight cuproptosis-related genes (FDX1, DLD, DBT, GCSH, DLAT, SLC31A1, ATP7A, and ATP7B) with prognostic significance (Figure 3(c)). Further screening was then performed using LASSO regression, and four genes were identified to build the predictive model based on the optimal *λ* (lambda.min) (Figures 3(d) and 3(e)). The formula for the calculation of risk score is shown in detail: (0.950^∗^ FDX1) + (−1.328^∗^ GCSH) + (0.607^∗^ ATP7A) + (−1.495^∗^ ATP7B). The immune score was positively associated with the risk score, with higher risk score in the high immune score group (Figure 3(f)).

### 3.4. Internal and External Validation of Predictive Models

**Figure 4:**
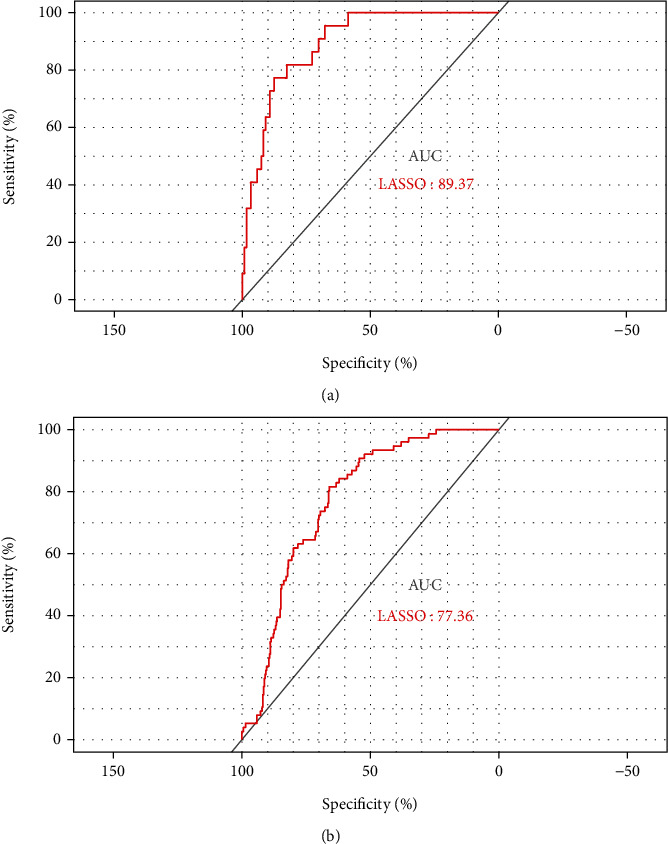
Internal and external validation of predictive models. (a) AUC of ROC curves in TCGA cohort (internal validation set). (b) AUC of ROC curves in CGGA cohort (external validation set).

Training and internal validation datasets were randomly generated from TCGA database's LGG cohort at a 7 : 3 ratio. The internal validation group has an AUC value of 0.8937 (Figure 4(a)), demonstrating a good predictive power. Without removing the batch effect, the external validation group's (CGGA) AUC was 0.7736 (Figure 4(b)), indicating that the model has good external predictive power.

### 3.5. Immune Cell Infiltration

**Figure 5:**
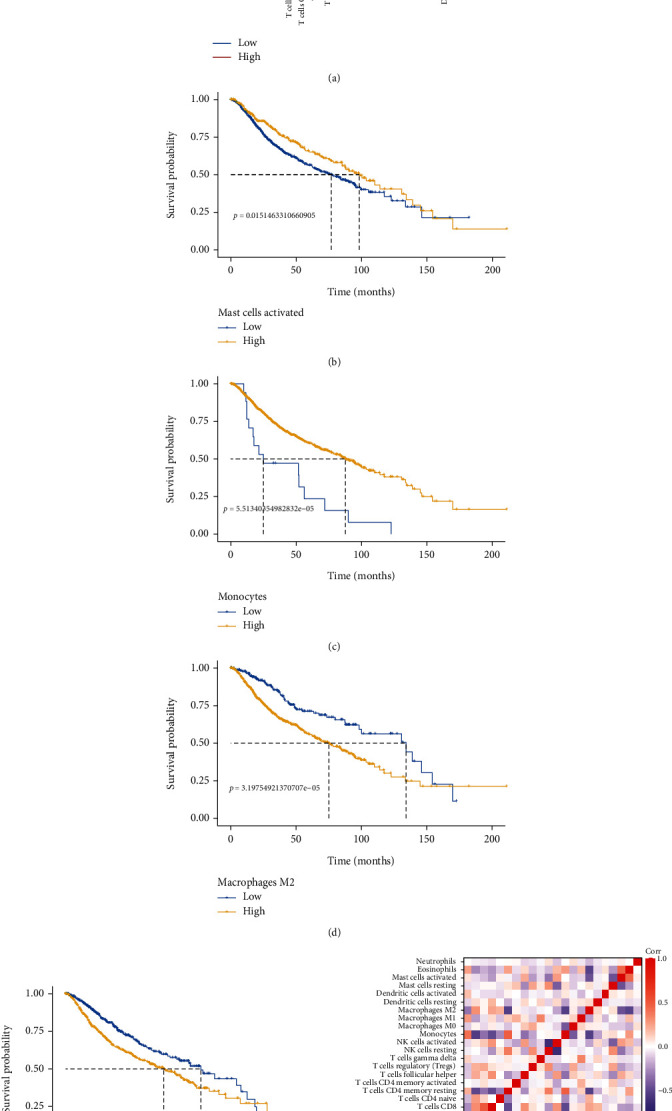
Demonstration of immune cell infiltration in LGG by CIBERSORT algorithm. (a) Violin plot comparing the proportion of infiltrating immune cells between LGG samples with low and high immune scores. Kaplan–Meier plot for the infiltration of (b) mast cells, (c) monocytes, (d) M2 macrophages, and (e) CD8 T cells and overall survival. (f) Association between different immune cells.

As shown by the vioplot in Figure 5(a), there were statistically significant differences in most of the 22 infiltrating immune cells between groups with high and low immune scores. M2-type macrophages were the most significantly enriched. Compared to the low immune score group, those with a high immune score had a greater number of memory B cells, CD8+ T cells, activated memory CD4+ T cells, regulatory T cells, M2-type macrophages, and resting mast cells. In contrast, the low immune score group had considerably larger amounts of naive B cells, resting memory CD4+ T cells, activated mast cells, eosinophils, and naive B cells. We further investigated the prognostic impact of these immune cells with significant differences in infiltration on LGG and ultimately found that activated mast cells (Figure 5(b)) and monocytes (Figure 5(c)) were prognostically beneficial, while M2 macrophages (Figure 5(d)) and CD8 T cells (Figure 5(e)) were prognostically detrimental. Subsequently, we performed correlation assays on 22 immune cells and found that M2 macrophages were negatively correlated with resting memory CD4 T cells, monocytes, and naive B cells and positively correlated with memory B cells and CD8 T cells (Figure 5(f)).

### 3.6. Correlation between Cuproptosis-Related Genes and Immunity

**Figure 6:**
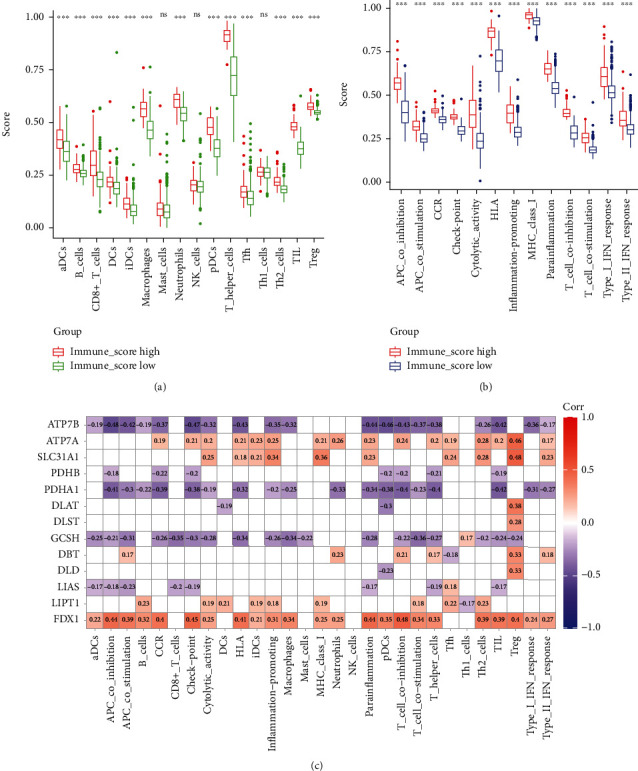
Comparison of the ssGSEA scores between different immune score groups in LGG. (a) The scores of 16 immune cells between different immune score groups in LGG. (b) The scores of 13 immune-related functions between different immune score groups in LGG. (c) Association between cuproptosis-related genes and immunity. Adjusted *p* values were showed as ns: not significant, ^∗^*p* < 0.05, ^∗∗^*p* < 0.01, and ^∗∗∗^*p* < 0.001.

Immune cell subpopulations and immune function enrichment varied significantly between the two distinct LGG immune score groups. As shown in the box plot (Figures 6(a) and 6(b)), the enrichment of 13 immune functions was significantly higher in the high immune score group, and the immune cells showed similar results. Subsequently, we did a correlation analysis of cuproptosis-related genes with immune cells and functions. As seen in the corrplot (Figure 6(c)), the four cuproptosis-related genes included in the prediction model were closely associated with immune regulation, with FDX1 and ATP7A showing positive regulatory relationships, while ATP7B and GCSH showed negative regulatory relationships.

### 3.7. TIDE Score

**Figure 7:**
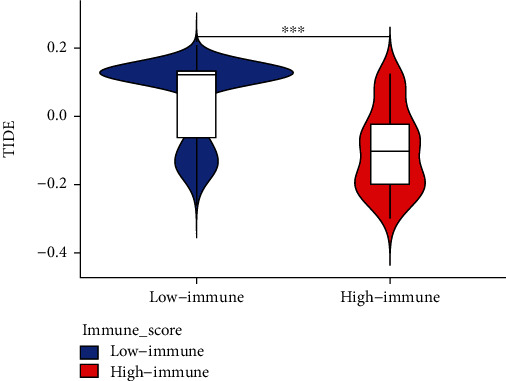
Comparison of the TIDE scores between different immune score groups in LGG.

Finally, we used the TIDE algorithm to obtain scores of immune escape ability in disparate immune-score LGG patients (Supplementary Material 2). As shown in the vioplot (Figure 7), significantly higher TIDE scores were seen in the low immune group compared to the high immune group, suggesting that the LGG population at high risk, the group with the high immune score, had a low immune escape potential.

## 4. Discussion

In this study, we separated LGG into groups with high and low immune scores and discovered that patients with a high immune score had a shorter OS. Subsequently, we constructed a concise and efficient prediction model based on cuproptosis-related genes for the first time, which can better identify LGG patients with high immune scores in both the internal dataset (TCGA) and the external dataset (CGGA). Moreover, we found a very different immune landscape in varied risk LGG patients. The cuproptosis-related genes involved in the construction of the prediction model were found to be closely related to immune function by correlation analysis.

The immune score responds to the degree of immune cell infiltration around the tumor and also to the level of immunity. Our study showed that LGG patients in the group with a high immune score had a worse prognosis, which is consistent with the results of a previous study [5]. Cuproptosis has just recently been proposed as a new mode of cell death, and there is still a brand new field of research on the relevance of genes associated with cuproptosis to the OS of LGG patients. Interestingly, significant differences in the expression of most cuproptosis-related genes were found between LGG groups with high and low immune scores (Figures 3(a) and 3(b)), and eight cuproptosis-related genes were connected to OS in a univariate Cox analysis. These results suggest the involvement of cuproptosis in LGG progression and the possibility of building predictive models with these cuproptosis-related genes.

This study's predictive model was composed of four cuproptosis-related genes (FDX1, GCSH, ATP7A, and ATP7B). FDX1 is a key gene mediating cuproptosis. FDX1 converts Cu2+ to the more toxic Cu+, which can contribute to aberrant oligomerization of thioctylated proteins in the TCA cycle, and FDX1 destabilises Fe-S cluster proteins [8]. Glycine cleavage system protein H (GCSH) is central to this system through its lipid acyl arm that functionally connects all other glycine cleavage system enzymes [16]. In addition, GCSH is a lipid acylated modifier protein, an essential component of the PDH complex that regulates the entry of carbon into the tricarboxylic acid cycle and is essential for the induction of cuproptosis [8, 17]. ATP7A and ATP7B are copper transport proteins responsible for copper transfer out and maintaining intracellular copper homeostasis. If intracellular copper homeostasis is dysregulated, an excess of intracellular copper will induce cuproptosis [8, 18]. In conclusion, two genes (ATP7A, ATP7B) in the prediction model can protect cells from cuproptosis, while two genes (FDX1, GCSH) are opposite.

Therefore, from the perspective of cuproptosis alone, FDX1 and GCSH should be the protective factors for LGG patients, while ATP7A and ATP7B are the opposite. However, we found that FDX1 and ATP7A were prognostic risk-related genes, while GCSH and ATP7B were protective genes by Cox regression analysis. Why is the theoretical inference not entirely consistent with our actual results? Let us return to the LGG risk stratification. Most cuproptosis-related genes (including the key gene FDX1) were expressed higher in the high immune score group than in the low immune score group, yet the prognosis of patients in the high immune group was significantly worse than in the low immune score group. This suggested that we need to consider immune factors in addition to cuproptosis to influence the prognosis of LGG. Unlike other tumors, gliomas with a strong immune response have a poorer prognosis [5]. Recent studies have found that M2 macrophages are significantly enriched in LGG, with a proportion of up to 30-50% in the glioma microenvironment [19]. The prognostic role of immune cells has also been reported, and the current study suggests that M2 macrophages are a poor prognostic factor in glioma [20]. Our results also showed that M2 macrophages accounted for the highest proportion of 22 immune cells, and they were significantly higher in the high immune score group than in the low immune score group. This answered our previous suspicion. Therefore, it was reasonable to speculate that cuproptosis might strengthen the local immune response.

We believed that these cuproptosis-related genes might play a role in immunological modulation in addition to the regulation of cuproptosis. The previous correlation analysis also proved our view that FDX1 and ATP7A showed a significant positive correlation with immune function, while GCSH and ATP7B showed the opposite. FDX1 is a mitochondrial electron transport chain- (ETC-) associated gene that participates in the reduction of mitochondrial cytochromes and serves a crucial function in energy metabolism [21]. The initiation of antitumor immune response undoubtedly requires the involvement of energy metabolism [22]. It had been found that FDX1 was closely associated with the infiltration of a variety of immune cells, and in particular, it showed a high positive correlation with macrophage infiltration [23]. ATP7A and ATP7B are both copper transport proteins, but their functions are not the same. ATP7A is significantly more efficient than ATP7B in transporting copper and is the main force of intracellular copper excretion, while ATP7B is closely related to the synthesis of copper cyanobactin [24–26]. Lower copper levels have been shown to result in lower levels of bone marrow-derived suppressor cells and enhanced immune responses [27]. As a ferroxidase, ceruloplasmin is also associated with tumor progression. In the study of breast cancer, it was discovered that the level of ceruloplasmin expression was closely connected to immune cell infiltration, and M2 macrophages were negatively correlated with ceruloplasmin [28]. However, so far, we had found no studies on GCSH related to immunity. To sum up, FDX1 and ATP7A could promote the infiltration of M2 macrophages, while ATP7B was the opposite. Taken together, we believed that most cuproptosis-related genes were highly expressed in the LGG high immune score group, thus enhancing the local immune response of tumors. Nevertheless, glioma with high immune status was an unfavourable prognostic factor, and the final result was to cover up the beneficial effect of cuproptosis on LGG.

Gliomas were considered to be “cold tumors,” and it was now believed that “hot” tumors are more likely to respond to immunotherapy [19, 29]. However, high-immune LGG patients demonstrated weaker immune escape than low-immune individuals, which might indicate that this high-risk group could benefit from immunotherapy. Therefore, it was necessary to use our prediction model to select high-risk LGG patients for prospective studies of immunotherapy in the future.

There are also caveats to this study. First, the patient data we used are retrospective data from public databases, and it is necessary for our model to be validated in future prospective trials. However, this study also has its unique advantages. Our model is based on TCGA and has strong prediction power in the CGGA database without removing batch effects. This demonstrates that our model has good clinical application across different racial groups. Second, using a single gene set to construct a prediction model inevitably excludes some genes that affect the prognosis of LGG. However, our study is the first to point out that cuproptosis-related genes are closely associated with the prognosis of LGG. Finally, the conclusions of this investigation need to be further demonstrated by in vivo and in vitro experiments.

## 5. Conclusions

In this study, the prediction model based on four cuproptosis-related genes could accurately identify the high-risk LGG patients. High expression of cuproptosis-related genes might lead to high immune response and poor prognosis in LGG patients, but such high-risk populations might benefit from immunotherapy. In this study, we took a first pass at dissecting the connection between cuproptosis-related genes and the tumor immune microenvironment in LGG, but the specific mechanism remains poorly understood and deserves further study.

## Figures and Tables

**Table 1:** Clinical characteristic of the LGG patient used in this study.

	TCGA		CGGA		Total	
	Low immune score (*N* = 438)	High immune score (*N* = 68)	Low immune score (*N* = 344)	High immune score (*N* = 76)	Low immune score (*N* = 782)	High immune score (*N* = 144)
Sex						
Female	196 (44.7%)	31 (45.6%)	151 (43.9%)	34 (44.7%)	347 (44.4%)	65 (45.1%)
Male	242 (55.3%)	37 (54.4%)	193 (56.1%)	42 (55.3%)	435 (55.6%)	79 (54.9%)
Age (years)						
Median [min, max]	41.0 [14.0, 87.0]	44.0 [22.0, 75.0]	40.0 [12.0, 69.0]	40.0 [11.0, 72.0]	41.0 [12.0, 87.0]	41.0 [11.0, 75.0]
Status						
Alive	341 (77.9%)	39 (57.4%)	193 (56.1%)	30 (39.5%)	534 (68.3%)	69 (47.9%)
Dead	97 (22.1%)	29 (42.6%)	151 (43.9%)	46 (60.5%)	248 (31.7%)	75 (52.1%)
Grade						
II	232 (53.0%)	14 (20.6%)	144 (41.9%)	28 (36.8%)	376 (48.1%)	42 (29.2%)
Ill	206 (47.0%)	54 (79.4%)	200 (58.1%)	48 (63.2%)	406 (51.9%)	102 (70.8%)
Follow-up time (months)						
^Median^ [min, max]	22.3 [0.0300, 211]	21.5 [0.100, 105]	50.6 ^[1.70, 158]^	33.3 ^[2.23, 168]^	31.2 ^[0.0300, 211]^	^26.6 [0^.^100, 168]^

## Data Availability

All data, models, and code generated or used during the study appear in the submitted article.

## Supplementary Materials

Supplementary Materials Supplementary Figure 1: X-tile plots: cutoff values for immune scores. Supplementary Material 2: TIDE score.
