# Supplementary material for: Novel Cuproptosis-Related Gene Signature for Precise Identification of High-Risk Populations in Low-Grade Gliomas
Source: Mediators Inflamm. 2023 Feb 13;2023:6232620. doi: 10.1155/2023/6232620 (PMC9940981; doi:10.1155/2023/6232620)
Supplement: Supplementary Materials — Supplementary Figure 1: X-tile plots: cutoff values for immune scores. Supplementary Material 2: TIDE score. [file 6232620.f1.zip › Supplementary Material 2.pdf]

	Dysfunction	Exclusion	status	TIDE
TCGA-CS-4938	-0.1433904	0.123176752	Low	0.123177
TCGA-CS-4941	-0.0597118	0.110553197	High	-0.05971
TCGA-CS-4942	-0.1775445	0.098107284	Low	0.098107
TCGA-CS-4943	-0.2365248	0.130216937	Low	0.130217
TCGA-CS-4944	-0.1674914	0.130706208	High	-0.16749
TCGA-CS-5390	-0.1222794	0.138844023	High	-0.12228
TCGA-CS-5393	-0.2017281	0.108867236	Low	0.108867
TCGA-CS-5394	-0.0533207	0.138212149	Low	0.138212
TCGA-CS-5395	-0.0483717	0.146615506	Low	0.146616
TCGA-CS-5396	-0.0538741	0.14158645	High	-0.05387
TCGA-CS-5397	-0.0593364	0.14826726	Low	0.148267
TCGA-CS-6186	-0.087222	0.120888269	Low	0.120888
TCGA-CS-6188	-0.0498815	0.151503265	Low	0.151503
TCGA-CS-6290	-0.1876589	0.091237677	High	-0.18766
TCGA-CS-6666	-0.1787164	0.141367615	High	-0.17872
TCGA-CS-6667	-0.1717511	0.126042286	Low	0.126042
TCGA-CS-6668	-0.0681137	0.127123081	Low	0.127123
TCGA-CS-6669	-0.1166974	0.12052842	Low	0.120528
TCGA-CS-6670	-0.1312359	0.134551638	Low	0.134552
TCGA-DB-5270	-0.1542581	0.125042337	High	-0.15426
TCGA-DB-5273	-0.1969676	0.128103499	Low	0.128103
TCGA-DB-5274	-0.2190312	0.135488486	High	-0.21903
TCGA-DB-5275	-0.2100883	0.128141109	Low	0.128141
TCGA-DB-5277	-0.1953845	0.141987633	Low	0.141988
TCGA-DB-5278	-0.0988971	0.131793327	Low	0.131793
TCGA-DB-5279	-0.0783371	0.123602113	High	-0.07834
TCGA-DB-5280	-0.1583434	0.129062916	Low	0.129063
TCGA-DB-5281	-0.1490884	0.144237507	High	-0.14909
TCGA-DB-A4X9	-0.1713655	0.123841604	Low	0.123842
TCGA-DB-A4XA	-0.1318726	0.122754943	High	-0.13187
TCGA-DB-A4XB	-0.1878412	0.129017745	Low	0.129018
TCGA-DB-A4XC	-0.1653095	0.116931137	Low	0.116931
TCGA-DB-A4XD	-0.1762644	0.129470496	Low	0.12947
TCGA-DB-A4XE	-0.1768733	0.136942255	Low	0.136942
TCGA-DB-A4XF	-0.1582712	0.134519237	Low	0.134519
TCGA-DB-A4XG	-0.0984682	0.149229232	Low	0.149229
TCGA-DB-A4XH	-0.1221462	0.136066726	Low	0.136067
TCGA-DB-A64L	-0.1176208	0.103650638	High	-0.11762
TCGA-DB-A64O	-0.0688263	0.130719332	Low	0.130719
TCGA-DB-A64P	-0.1164834	0.136572941	Low	0.136573
TCGA-DB-A64Q	-0.0616578	0.129640919	Low	0.129641
TCGA-DB-A64R	-0.1052118	0.135425289	Low	0.135425
TCGA-DB-A64S	-0.1897348	0.112963889	Low	0.112964
TCGA-DB-A64U	-0.1187788	0.127898418	Low	0.127898
TCGA-DB-A64V	-0.1620133	0.103684769	High	-0.16201
TCGA-DB-A64W	-0.1456606	0.156920407	Low	0.15692
TCGA-DB-A64X	-0.2407864	0.137339003	Low	0.137339
TCGA-DB-A75K	-0.1693962	0.114044268	Low	0.114044
TCGA-DB-A75L	-0.2004166	0.132078785	Low	0.132079
TCGA-DB-A75M	-0.2246177	0.123037136	Low	0.123037
TCGA-DB-A75O	-0.2350536	0.11081377	Low	0.110814
TCGA-DB-A75P	-0.1299398	0.139395838	High	-0.12994
TCGA-DH-5140	-0.1328438	0.165478305	High	-0.13284
TCGA-DH-5141	-0.1252631	0.130523861	Low	0.130524
TCGA-DH-5142	-0.2448873	0.116923063	High	-0.24489
TCGA-DH-5143	-0.186088	0.119100617	Low	0.119101
TCGA-DH-5144	-0.0883034	0.125865556	Low	0.125866

TCGA-DH-A669	-0.1455054	0.139469834	High	-0.14551
TCGA-DH-A66B	-0.2468165	0.116400116	High	-0.24682
TCGA-DH-A66D	-0.2292803	0.112946343	Low	0.112946
TCGA-DH-A66F	-0.1061775	0.128213734	Low	0.128214
TCGA-DH-A66G	-0.1421621	0.124334858	High	-0.14216
TCGA-DH-A7UR	-0.1421255	0.145638781	High	-0.14213
TCGA-DH-A7US	-0.1396259	0.129009853	Low	0.12901
TCGA-DH-A7UT	-0.2385046	0.122797667	Low	0.122798
TCGA-DH-A7UU	-0.2319243	0.131035787	Low	0.131036
TCGA-DH-A7UV	-0.2227064	0.108882634	Low	0.108883
TCGA-DU-5847	-0.0595122	0.125241211	High	-0.05951
TCGA-DU-5849	-0.1263755	0.125272455	High	-0.12638
TCGA-DU-5852	-0.0832401	0.14613846	High	-0.08324
TCGA-DU-5853	-0.1859323	0.1081687	High	-0.18593
TCGA-DU-5854	-0.0594693	0.134165252	High	-0.05947
TCGA-DU-5855	-0.202009	0.116449363	Low	0.116449
TCGA-DU-5870	-0.0601432	0.131900997	Low	0.131901
TCGA-DU-5871	-0.1979498	0.111234673	Low	0.111235
TCGA-DU-5872	-0.1389496	0.09689524	High	-0.13895
TCGA-DU-5874	-0.1123397	0.141901888	Low	0.141902
TCGA-DU-6392	-0.26086	0.145233322	High	-0.26086
TCGA-DU-6393	-0.1329747	0.143506365	Low	0.143506
TCGA-DU-6394	-0.0992599	0.13649346	Low	0.136493
TCGA-DU-6395	-0.2199914	0.121798923	Low	0.121799
TCGA-DU-6396	-0.2245847	0.094725741	High	-0.22458
TCGA-DU-6397	-0.1200361	0.159273733	High	-0.12004
TCGA-DU-6399	-0.1866842	0.128588728	Low	0.128589
TCGA-DU-6400	-0.1425919	0.122230926	High	-0.14259
TCGA-DU-6401	-0.1803574	0.124638063	Low	0.124638
TCGA-DU-6402	-0.0020366	0.155458428	High	-0.00204
TCGA-DU-6403	-0.1401485	0.125907168	Low	0.125907
TCGA-DU-6404	-0.0439108	0.208620633	Low	0.208621
TCGA-DU-6405	-0.0656151	0.147315398	Low	0.147315
TCGA-DU-6406	-0.0708804	0.150777654	High	-0.07088
TCGA-DU-6407	-0.2575026	0.136634347	Low	0.136634
TCGA-DU-6408	-0.2137481	0.128174114	Low	0.128174
TCGA-DU-6410	-0.1161904	0.146746257	Low	0.146746
TCGA-DU-6542	-0.1849894	0.12312903	High	-0.18499
TCGA-DU-7006	0.01461669	0.149052712	High	0.014617
TCGA-DU-7007	-0.1628528	0.136860697	High	-0.16285
TCGA-DU-7008	-0.2075506	0.134399456	Low	0.134399
TCGA-DU-7009	-0.0921415	0.134747057	Low	0.134747
TCGA-DU-7010	-0.1891108	0.151539234	Low	0.151539
TCGA-DU-7011	-0.1301062	0.122983467	High	-0.13011
TCGA-DU-7012	0.03361555	0.117754351	High	0.033616
TCGA-DU-7013	-0.0291814	0.144103225	High	-0.02918
TCGA-DU-7014	-0.1908359	0.134548244	Low	0.134548
TCGA-DU-7015	-0.1697654	0.107177494	High	-0.16977
TCGA-DU-7018	-0.1002436	0.152822233	Low	0.152822
TCGA-DU-7290	0.02373703	0.105944144	High	0.023737
TCGA-DU-7292	-0.0833416	0.155425829	High	-0.08334
TCGA-DU-7294	-0.0768163	0.134933958	Low	0.134934
TCGA-DU-7298	-0.1735685	0.126389709	Low	0.12639
TCGA-DU-7299	-0.1908371	0.109526309	Low	0.109526
TCGA-DU-7300	-0.0796578	0.128510737	High	-0.07966
TCGA-DU-7301	-0.1787979	0.124417286	Low	0.124417
TCGA-DU-7302	-0.0604178	0.133287269	Low	0.133287
TCGA-DU-7304	-0.1588847	0.11563935	High	-0.15888

TCGA-DU-7306	-0.1427455	0.118092569	Low	0.118093
TCGA-DU-7309	-0.143453	0.129082942	Low	0.129083
TCGA-DU-8158	0.01012588	0.126505485	High	0.010126
TCGA-DU-8161	-0.0044536	0.110654416	High	-0.00445
TCGA-DU-8162	-0.0834087	0.126863959	Low	0.126864
TCGA-DU-8163	-0.1574763	0.113693772	High	-0.15748
TCGA-DU-8164	-0.0730709	0.135901219	Low	0.135901
TCGA-DU-8165	0.00398697	0.134367242	High	0.003987
TCGA-DU-8166	-0.1465665	0.114940358	Low	0.11494
TCGA-DU-8167	-0.2216948	0.111912939	Low	0.111913
TCGA-DU-8168	-0.0939989	0.13901341	Low	0.139013
TCGA-DU-A5TP	-0.208639	0.100909631	High	-0.20864
TCGA-DU-A5TR	-0.277117	0.095618217	High	-0.27712
TCGA-DU-A5TS	-0.2587877	0.123683654	Low	0.123684
TCGA-DU-A5TT	-0.0856129	0.13337954	Low	0.13338
TCGA-DU-A5TU	-0.2603599	0.124443199	Low	0.124443
TCGA-DU-A5TW	-0.1959662	0.130894884	Low	0.130895
TCGA-DU-A5TY	-0.0619696	0.119162131	High	-0.06197
TCGA-DU-A6S2	-0.1128649	0.121040776	High	-0.11286
TCGA-DU-A6S3	-0.1752926	0.140824553	Low	0.140825
TCGA-DU-A6S6	-0.1435608	0.141190991	Low	0.141191
TCGA-DU-A6S7	-0.2076202	0.115043874	Low	0.115044
TCGA-DU-A6S8	-0.1293086	0.135408417	High	-0.12931
TCGA-DU-A76K	-0.1150784	0.157983711	High	-0.11508
TCGA-DU-A76L	-0.0280751	0.151304545	High	-0.02808
TCGA-DU-A76O	-0.2444903	0.111290464	Low	0.11129
TCGA-DU-A76R	-0.0551732	0.143351848	Low	0.143352
TCGA-DU-A7T6	-0.1530563	0.165438731	High	-0.15306
TCGA-DU-A7T8	-0.2104336	0.133648023	High	-0.21043
TCGA-DU-A7TA	-0.2038719	0.124244208	High	-0.20387
TCGA-DU-A7TB	-0.15058	0.130661469	Low	0.130661
TCGA-DU-A7TC	-0.236461	0.132946043	Low	0.132946
TCGA-DU-A7TD	-0.0603372	0.121390117	High	-0.06034
TCGA-DU-A7TG	-0.12579	0.116939128	Low	0.116939
TCGA-DU-A7TI	-0.1847543	0.103268917	Low	0.103269
TCGA-DU-A7TJ	-0.0567716	0.155017708	High	-0.05677
TCGA-E1-5302	-0.1837704	0.120788402	Low	0.120788
TCGA-E1-5303	-0.2246286	0.108459675	Low	0.10846
TCGA-E1-5304	-0.2808204	0.153256337	Low	0.153256
TCGA-E1-5305	-0.1768018	0.133226419	Low	0.133226
TCGA-E1-5307	-0.1965243	0.112992705	Low	0.112993
TCGA-E1-5311	-0.1121226	0.139086124	Low	0.139086
TCGA-E1-5318	-0.1246535	0.138532157	Low	0.138532
TCGA-E1-5319	-0.0792421	0.126902266	Low	0.126902
TCGA-E1-5322	-0.2239459	0.114116244	Low	0.114116
TCGA-E1-A7YD	-0.0614426	0.151863197	High	-0.06144
TCGA-E1-A7YE	-0.2003277	0.124960135	High	-0.20033
TCGA-E1-A7YH	-0.2272209	0.120109703	Low	0.12011
TCGA-E1-A7YI	-0.202176	0.157542277	Low	0.157542
TCGA-E1-A7YJ	-0.1030913	0.121962351	High	-0.10309
TCGA-E1-A7YK	-0.2166784	0.106940579	High	-0.21668
TCGA-E1-A7YL	-0.0824472	0.14252726	High	-0.08245
TCGA-E1-A7YM	-0.1263134	0.132878076	Low	0.132878
TCGA-E1-A7YN	-0.0774862	0.070165407	High	-0.07749
TCGA-E1-A7YO	-0.0984161	0.139963182	Low	0.139963
TCGA-E1-A7YQ	-0.0628047	0.128635079	High	-0.0628
TCGA-E1-A7YS	-0.1440935	0.131287158	Low	0.131287
TCGA-E1-A7YU	-0.2457997	0.129546769	Low	0.129547

TCGA-E1-A7YV	-0.1680472	0.166583507	High	-0.16805
TCGA-E1-A7YW	-0.2368167	0.122729315	Low	0.122729
TCGA-E1-A7YY	-0.1408605	0.11824053	Low	0.118241
TCGA-E1-A7Z2	-0.1649723	0.147015943	Low	0.147016
TCGA-E1-A7Z3	-0.1550582	0.129655095	High	-0.15506
TCGA-E1-A7Z4	-0.2135517	0.104291118	Low	0.104291
TCGA-E1-A7Z6	-0.1498431	0.11880784	Low	0.118808
TCGA-EZ-7264	-0.0713088	0.134078893	Low	0.134079
TCGA-F6-A8O3	-0.1231774	0.139608082	Low	0.139608
TCGA-F6-A8O4	-0.1781432	0.137916992	Low	0.137917
TCGA-FG-5962	-0.0908401	0.127954621	High	-0.09084
TCGA-FG-5963	-0.161501	0.119808597	High	-0.1615
TCGA-FG-5964	-0.1665621	0.113199106	High	-0.16656
TCGA-FG-5965	-0.1658972	0.139492479	Low	0.139492
TCGA-FG-6688	-0.0742662	0.118860424	Low	0.11886
TCGA-FG-6689	-0.2023998	0.110121173	Low	0.110121
TCGA-FG-6690	-0.1628556	0.110124837	Low	0.110125
TCGA-FG-6691	-0.1280358	0.103122788	Low	0.103123
TCGA-FG-6692	-0.061023	0.149088385	High	-0.06102
TCGA-FG-7634	-0.0823117	0.136247519	High	-0.08231
TCGA-FG-7636	-0.2286924	0.12742934	Low	0.127429
TCGA-FG-7637	-0.0758412	0.144660424	Low	0.14466
TCGA-FG-7638	-0.1091332	0.140056193	High	-0.10913
TCGA-FG-7641	-0.0954822	0.130783062	Low	0.130783
TCGA-FG-7643	-0.0802422	0.13141993	High	-0.08024
TCGA-FG-8181	-0.1002817	0.122606249	High	-0.10028
TCGA-FG-8182	-0.1632249	0.105536141	Low	0.105536
TCGA-FG-8185	-0.2284601	0.134163473	Low	0.134163
TCGA-FG-8186	-0.1056633	0.10779112	High	-0.10566
TCGA-FG-8187	-0.1121809	0.116832962	Low	0.116833
TCGA-FG-8188	-0.1726095	0.120036526	Low	0.120037
TCGA-FG-8189	-0.1110512	0.124689118	Low	0.124689
TCGA-FG-8191	-0.140236	0.112515912	Low	0.112516
TCGA-FG-A4MT	-0.2247404	0.138609158	Low	0.138609
TCGA-FG-A4MU	-0.0592513	0.149343125	High	-0.05925
TCGA-FG-A4MW	-0.131799	0.157562035	Low	0.157562
TCGA-FG-A4MX	-0.2388924	0.127235182	Low	0.127235
TCGA-FG-A4MY	-0.2230317	0.129277852	Low	0.129278
TCGA-FG-A60J	-0.1896793	0.121865921	Low	0.121866
TCGA-FG-A60K	-0.0874631	0.126802454	Low	0.126802
TCGA-FG-A60L	-0.300231	0.110745615	Low	0.110746
TCGA-FG-A6IZ	-0.2301495	0.129714381	Low	0.129714
TCGA-FG-A6J1	-0.2293819	0.128937209	High	-0.22938
TCGA-FG-A6J3	-0.2988807	0.141055308	High	-0.29888
TCGA-FG-A70Y	-0.2271949	0.115049716	Low	0.11505
TCGA-FG-A70Z	-0.1049473	0.149837902	High	-0.10495
TCGA-FG-A710	-0.1489737	0.115607718	High	-0.14897
TCGA-FG-A711	-0.2283157	0.125267161	Low	0.125267
TCGA-FG-A713	-0.1381802	0.133965316	Low	0.133965
TCGA-FG-A87N	-0.1657125	0.15776053	Low	0.157761
TCGA-FG-A87Q	-0.1047266	0.110313771	High	-0.10473
TCGA-FN-7833	-0.1711384	0.094128713	Low	0.094129
TCGA-HT-7467	-0.1136333	0.130365905	Low	0.130366
TCGA-HT-7468	-0.0632561	0.136079738	Low	0.13608
TCGA-HT-7469	-0.1637842	0.144319033	Low	0.144319
TCGA-HT-7470	-0.1720899	0.127085251	Low	0.127085
TCGA-HT-7471	-0.1051169	0.128759661	High	-0.10512
TCGA-HT-7472	-0.1482413	0.118759296	Low	0.118759

TCGA-HT-7473	-0.1211306	0.086027794	High	-0.12113
TCGA-HT-7474	-0.1434057	0.125893182	High	-0.14341
TCGA-HT-7475	-0.1872687	0.135439585	Low	0.13544
TCGA-HT-7476	-0.1584165	0.14873569	High	-0.15842
TCGA-HT-7477	-0.2159969	0.150749235	Low	0.150749
TCGA-HT-7478	-0.1848236	0.102056595	High	-0.18482
TCGA-HT-7479	-0.1585443	0.109247556	Low	0.109248
TCGA-HT-7480	-0.0863976	0.135755308	Low	0.135755
TCGA-HT-7481	-0.09702	0.133152421	Low	0.133152
TCGA-HT-7482	-0.1593305	0.117006507	Low	0.117007
TCGA-HT-7483	-0.1421201	0.146589248	High	-0.14212
TCGA-HT-7485	-0.1300334	0.113237399	Low	0.113237
TCGA-HT-7601	-0.1766181	0.106577355	High	-0.17662
TCGA-HT-7602	-0.1338622	0.111788964	Low	0.111789
TCGA-HT-7603	-0.1748117	0.12786865	Low	0.127869
TCGA-HT-7604	-0.1788998	0.133715541	Low	0.133716
TCGA-HT-7605	-0.1052484	0.123523372	Low	0.123523
TCGA-HT-7606	-0.2009099	0.144703249	Low	0.144703
TCGA-HT-7607	-0.0801828	0.125218648	High	-0.08018
TCGA-HT-7608	-0.087892	0.122400661	Low	0.122401
TCGA-HT-7609	-0.19912	0.114527984	Low	0.114528
TCGA-HT-7610	-0.1301081	0.123928334	Low	0.123928
TCGA-HT-7611	-0.1632527	0.115227286	Low	0.115227
TCGA-HT-7616	-0.0088917	0.104973103	High	-0.00889
TCGA-HT-7620	-0.1068526	0.111382583	Low	0.111383
TCGA-HT-7676	-0.2071484	0.110099599	Low	0.1101
TCGA-HT-7677	-0.0807592	0.127659564	High	-0.08076
TCGA-HT-7680	-0.045598	0.111824417	Low	0.111824
TCGA-HT-7681	-0.1258954	0.127851878	High	-0.1259
TCGA-HT-7684	-0.2124863	0.115803791	Low	0.115804
TCGA-HT-7686	-0.2167411	0.106627292	High	-0.21674
TCGA-HT-7687	-0.0837672	0.124542416	High	-0.08377
TCGA-HT-7688	-0.1498325	0.127677786	High	-0.14983
TCGA-HT-7689	-0.1611921	0.121495311	Low	0.121495
TCGA-HT-7690	-0.2206683	0.128717046	High	-0.22067
TCGA-HT-7691	-0.0798133	0.12562257	High	-0.07981
TCGA-HT-7692	-0.1340627	0.130549192	Low	0.130549
TCGA-HT-7693	-0.1879031	0.112287952	High	-0.1879
TCGA-HT-7694	-0.1104799	0.125712416	Low	0.125712
TCGA-HT-7695	-0.0909681	0.131471041	Low	0.131471
TCGA-HT-7854	-0.0959923	0.097247763	High	-0.09599
TCGA-HT-7855	-0.1970029	0.121512561	Low	0.121513
TCGA-HT-7856	-0.1295003	0.129277751	Low	0.129278
TCGA-HT-7857	-0.0831043	0.123369336	High	-0.0831
TCGA-HT-7858	-0.1753504	0.102814522	High	-0.17535
TCGA-HT-7860	-0.0165707	0.119007536	High	-0.01657
TCGA-HT-7873	-0.1963601	0.121399001	Low	0.121399
TCGA-HT-7874	-0.0849401	0.12515872	Low	0.125159
TCGA-HT-7875	-0.0806123	0.145603867	Low	0.145604
TCGA-HT-7877	-0.0849217	0.134376153	Low	0.134376
TCGA-HT-7879	-0.1920843	0.123329879	Low	0.12333
TCGA-HT-7880	-0.1580441	0.124451114	High	-0.15804
TCGA-HT-7881	-0.1080011	0.129346853	Low	0.129347
TCGA-HT-7882	0.04972139	0.139653222	High	0.049721
TCGA-HT-7884	-0.1746192	0.127412257	Low	0.127412
TCGA-HT-7902	-0.1652055	0.122223989	High	-0.16521
TCGA-HT-8010	-0.0907579	0.122684805	Low	0.122685
TCGA-HT-8011	-0.0917321	0.161212263	High	-0.09173

TCGA-HT-8012	-0.1360987	0.121503058	Low	0.121503
TCGA-HT-8013	-0.2141	0.104901956	Low	0.104902
TCGA-HT-8015	-0.1301048	0.119012727	High	-0.1301
TCGA-HT-8018	-0.1262479	0.123347248	High	-0.12625
TCGA-HT-8019	-0.1455913	0.127721649	Low	0.127722
TCGA-HT-8104	-0.0573993	0.153576575	High	-0.0574
TCGA-HT-8105	-0.070713	0.121529523	High	-0.07071
TCGA-HT-8107	-0.1076175	0.111236649	Low	0.111237
TCGA-HT-8108	-0.1604957	0.104274922	Low	0.104275
TCGA-HT-8109	-0.0820609	0.116685243	Low	0.116685
TCGA-HT-8110	-0.028857	0.103423247	High	-0.02886
TCGA-HT-8111	-0.175112	0.115260017	Low	0.11526
TCGA-HT-8113	-0.1220072	0.121652833	High	-0.12201
TCGA-HT-8114	-0.1534646	0.119278181	High	-0.15346
TCGA-HT-8558	-0.1331527	0.119172634	Low	0.119173
TCGA-HT-8563	-0.1622456	0.093404638	High	-0.16225
TCGA-HT-8564	-0.1187238	0.137673726	High	-0.11872
TCGA-HT-A4DS	-0.2315507	0.125701317	High	-0.23155
TCGA-HT-A4DV	-0.1711141	0.11594164	High	-0.17111
TCGA-HT-A5R5	-0.1692677	0.108349006	Low	0.108349
TCGA-HT-A5R7	-0.1773554	0.127280985	Low	0.127281
TCGA-HT-A5R9	-0.1235939	0.152566966	High	-0.12359
TCGA-HT-A5RA	-0.013159	0.160103824	High	-0.01316
TCGA-HT-A5RB	-0.1792625	0.135645039	Low	0.135645
TCGA-HT-A5RC	-0.0496218	0.148985947	Low	0.148986
TCGA-HT-A614	-0.2112909	0.124827364	Low	0.124827
TCGA-HT-A615	-0.1193401	0.102044311	High	-0.11934
TCGA-HT-A616	-0.1671849	0.133122599	Low	0.133123
TCGA-HT-A617	-0.0626934	0.124652115	High	-0.06269
TCGA-HT-A618	-0.2121125	0.102477829	High	-0.21211
TCGA-HT-A619	-0.1259649	0.140128934	High	-0.12596
TCGA-HT-A61A	-0.1297101	0.127679229	High	-0.12971
TCGA-HT-A61B	-0.2473467	0.112305249	High	-0.24735
TCGA-HT-A61C	-0.1140702	0.153893427	High	-0.11407
TCGA-HT-A74H	-0.1157064	0.121666915	High	-0.11571
TCGA-HT-A74J	-0.2052288	0.124631592	Low	0.124632
TCGA-HT-A74K	-0.2183105	0.145642971	Low	0.145643
TCGA-HT-A74L	-0.0741515	0.121949649	Low	0.12195
TCGA-HT-A74O	-0.2807712	0.115484006	High	-0.28077
TCGA-HW-7486	-0.127172	0.114746306	Low	0.114746
TCGA-HW-7487	-0.108811	0.122274958	Low	0.122275
TCGA-HW-7489	-0.190975	0.126895071	Low	0.126895
TCGA-HW-7490	-0.1667952	0.12260051	High	-0.1668
TCGA-HW-7491	-0.0640249	0.133318736	Low	0.133319
TCGA-HW-7493	-0.1003374	0.119977332	High	-0.10034
TCGA-HW-7495	-0.0941065	0.133115118	Low	0.133115
TCGA-HW-8319	-0.1747433	0.128394814	Low	0.128395
TCGA-HW-8320	-0.1919033	0.119502806	Low	0.119503
TCGA-HW-8321	-0.169699	0.119459837	High	-0.1697
TCGA-HW-8322	-0.0795066	0.129454609	High	-0.07951
TCGA-HW-A5KJ	-0.1558363	0.145694752	High	-0.15584
TCGA-HW-A5KK	-0.1007169	0.104572925	High	-0.10072
TCGA-HW-A5KL	-0.18897	0.11910723	Low	0.119107
TCGA-HW-A5KM	-0.1924158	0.074221083	High	-0.19242
TCGA-IK-7675	-0.2000535	0.142230916	Low	0.142231
TCGA-IK-8125	-0.0828667	0.135983659	Low	0.135984
TCGA-KT-A74X	-0.1270795	0.132098772	High	-0.12708
TCGA-KT-A7W1	-0.0445523	0.157203051	High	-0.04455

TCGA-P5-A5EV	-0.1870276	0.122490303	High	-0.18703
TCGA-P5-A5EW	-0.184943	0.113419416	Low	0.113419
TCGA-P5-A5EX	-0.071805	0.118044003	High	-0.07181
TCGA-P5-A5EY	-0.1105844	0.129932985	High	-0.11058
TCGA-P5-A5EZ	-0.1955313	0.120689841	High	-0.19553
TCGA-P5-A5F0	-0.1102406	0.13748307	Low	0.137483
TCGA-P5-A5F1	-0.1799353	0.120815812	High	-0.17994
TCGA-P5-A5F2	-0.176027	0.126853057	Low	0.126853
TCGA-P5-A5F4	-0.1919855	0.13847552	High	-0.19199
TCGA-P5-A5F6	-0.0563389	0.120629412	Low	0.120629
TCGA-P5-A72U	-0.0462768	0.146820385	High	-0.04628
TCGA-P5-A72W	-0.2629983	0.131549382	Low	0.131549
TCGA-P5-A72X	-0.156572	0.138085504	High	-0.15657
TCGA-P5-A72Z	-0.1528835	0.14927177	Low	0.149272
TCGA-P5-A730	-0.1227762	0.150415975	Low	0.150416
TCGA-P5-A731	-0.1181018	0.151860056	Low	0.15186
TCGA-P5-A733	-0.1626781	0.133485495	Low	0.133485
TCGA-P5-A735	-0.2596143	0.123427814	Low	0.123428
TCGA-P5-A736	-0.1607987	0.10574147	High	-0.1608
TCGA-P5-A737	-0.1600275	0.130319677	Low	0.13032
TCGA-P5-A77W	-0.1227667	0.133895704	Low	0.133896
TCGA-P5-A77X	-0.0799092	0.13898002	Low	0.13898
TCGA-P5-A780	-0.192394	0.125794872	Low	0.125795
TCGA-P5-A781	-0.1082876	0.128844588	Low	0.128845
TCGA-QH-A65R	-0.1560658	0.140558481	Low	0.140558
TCGA-QH-A65S	-0.2136999	0.122317816	Low	0.122318
TCGA-QH-A65V	-0.1572642	0.124137685	Low	0.124138
TCGA-QH-A65X	-0.2018281	0.150007776	High	-0.20183
TCGA-QH-A65Z	-0.1383107	0.125414488	Low	0.125414
TCGA-QH-A6CS	-0.0677639	0.119158874	Low	0.119159
TCGA-QH-A6CU	-0.1467954	0.114333983	Low	0.114334
TCGA-QH-A6CV	-0.0923325	0.132746105	High	-0.09233
TCGA-QH-A6CW	-0.2292413	0.118329793	Low	0.11833
TCGA-QH-A6CX	-0.1029721	0.127172875	Low	0.127173
TCGA-QH-A6CY	-0.1249213	0.123949239	Low	0.123949
TCGA-QH-A6CZ	-0.1002016	0.128665811	Low	0.128666
TCGA-QH-A6X3	-0.1266836	0.127172826	Low	0.127173
TCGA-QH-A6X4	-0.150308	0.120802016	Low	0.120802
TCGA-QH-A6X5	-0.134248	0.128816654	Low	0.128817
TCGA-QH-A6X8	-0.1502426	0.119845343	Low	0.119845
TCGA-QH-A6X9	-0.1706578	0.125052056	Low	0.125052
TCGA-QH-A6XA	-0.181026	0.122846783	Low	0.122847
TCGA-QH-A6XC	-0.1441633	0.141842775	Low	0.141843
TCGA-QH-A86X	-0.0949318	0.134955298	Low	0.134955
TCGA-QH-A870	-0.1358347	0.120654209	Low	0.120654
TCGA-R8-A6MK	-0.0965491	0.133487083	Low	0.133487
TCGA-R8-A6ML	-0.0858396	0.137509174	Low	0.137509
TCGA-R8-A6MO	-0.1491272	0.120531965	High	-0.14913
TCGA-R8-A73M	-0.1876075	0.136950013	Low	0.13695
TCGA-RY-A83X	-0.1179813	0.134240445	Low	0.13424
TCGA-RY-A83Y	-0.164727	0.119820931	High	-0.16473
TCGA-RY-A83Z	-0.3055928	0.136647258	Low	0.136647
TCGA-RY-A840	-0.145633	0.142451937	Low	0.142452
TCGA-RY-A843	-0.1536864	0.12828698	Low	0.128287
TCGA-RY-A845	-0.1812525	0.125549011	Low	0.125549
TCGA-RY-A847	-0.1403912	0.114324806	Low	0.114325
TCGA-S9-A6TS	-0.2312281	0.125629589	Low	0.12563
TCGA-S9-A6TU	-0.226496	0.110662881	Low	0.110663

TCGA-S9-A6TV	-0.2293241	0.116911335	High	-0.22932
TCGA-S9-A6TW	-0.1046613	0.146665129	High	-0.10466
TCGA-S9-A6TX	-0.1226448	0.136018979	Low	0.136019
TCGA-S9-A6TY	-0.1120419	0.140506245	Low	0.140506
TCGA-S9-A6TZ	-0.140844	0.113736848	Low	0.113737
TCGA-S9-A6U0	-0.0606732	0.143073311	High	-0.06067
TCGA-S9-A6U1	-0.1958658	0.123084899	Low	0.123085
TCGA-S9-A6U2	-0.0635371	0.129201141	Low	0.129201
TCGA-S9-A6U5	-0.1226967	0.116451769	High	-0.1227
TCGA-S9-A6U6	-0.2005135	0.120821893	Low	0.120822
TCGA-S9-A6U8	-0.186004	0.113174331	Low	0.113174
TCGA-S9-A6U9	-0.171374	0.104198337	High	-0.17137
TCGA-S9-A6UA	-0.0732561	0.149356245	High	-0.07326
TCGA-S9-A6UB	-0.1271855	0.132693404	Low	0.132693
TCGA-S9-A6WD	-0.1276341	0.131670632	Low	0.131671
TCGA-S9-A6WE	-0.1253897	0.128636562	Low	0.128637
TCGA-S9-A6WG	-0.1647034	0.145333824	High	-0.1647
TCGA-S9-A6WH	-0.1407787	0.120290405	Low	0.12029
TCGA-S9-A6WI	-0.1639531	0.121951704	High	-0.16395
TCGA-S9-A6WL	-0.0816541	0.15515791	Low	0.155158
TCGA-S9-A6WM	-0.0355867	0.161591298	High	-0.03559
TCGA-S9-A6WN	-0.0609562	0.117462569	High	-0.06096
TCGA-S9-A6WO	-0.2020955	0.105845865	Low	0.105846
TCGA-S9-A6WP	-0.144283	0.117780544	High	-0.14428
TCGA-S9-A6WQ	-0.2290608	0.11331	Low	0.11331
TCGA-S9-A7IQ	-0.1374088	0.134661324	Low	0.134661
TCGA-S9-A7IS	-0.2184482	0.155117666	Low	0.155118
TCGA-S9-A7IX	-0.0904739	0.13638765	High	-0.09047
TCGA-S9-A7IY	-0.1374787	0.108075209	High	-0.13748
TCGA-S9-A7IZ	-0.1620052	0.12397857	Low	0.123979
TCGA-S9-A7J0	-0.232931	0.148114498	Low	0.148114
TCGA-S9-A7J1	-0.0758731	0.121166704	Low	0.121167
TCGA-S9-A7J2	-0.115881	0.137565145	Low	0.137565
TCGA-S9-A7J3	-0.1041383	0.111147903	Low	0.111148
TCGA-S9-A7QW	-0.2262546	0.138155579	Low	0.138156
TCGA-S9-A7QX	-0.2182141	0.121654978	Low	0.121655
TCGA-S9-A7QY	-0.1385728	0.130490813	Low	0.130491
TCGA-S9-A7QZ	-0.1290702	0.143467591	Low	0.143468
TCGA-S9-A7R1	-0.1308439	0.135378665	Low	0.135379
TCGA-S9-A7R2	-0.0703991	0.102427517	High	-0.0704
TCGA-S9-A7R3	-0.1576542	0.115851568	Low	0.115852
TCGA-S9-A7R4	-0.1514218	0.125422388	Low	0.125422
TCGA-S9-A7R7	-0.1461474	0.12763465	High	-0.14615
TCGA-S9-A7R8	-0.2254085	0.119347789	Low	0.119348
TCGA-S9-A89V	-0.1161541	0.130006297	High	-0.11615
TCGA-S9-A89Z	-0.2430392	0.130599992	High	-0.24304
TCGA-TM-A7C3	-0.0369021	0.156104713	High	-0.0369
TCGA-TM-A7C4	-0.1523318	0.137050414	Low	0.13705
TCGA-TM-A7C5	-0.1316158	0.133281215	Low	0.133281
TCGA-TM-A7CA	-0.2125566	0.1262825	Low	0.126283
TCGA-TM-A7CF	-0.201359	0.124932108	Low	0.124932
TCGA-TM-A84B	-0.0197632	0.151898732	High	-0.01976
TCGA-TM-A84C	-0.1025939	0.1523634	Low	0.152363
TCGA-TM-A84F	-0.2289108	0.092058735	High	-0.22891
TCGA-TM-A84G	-0.1008238	0.138065683	Low	0.138066
TCGA-TM-A84H	-0.1997318	0.121336898	Low	0.121337
TCGA-TM-A84I	-0.246384	0.14195135	Low	0.141951
TCGA-TM-A84J	-0.1727897	0.167878039	High	-0.17279

TCGA-TM-A84L	-0.1987795	0.11327963	Low	0.11328
TCGA-TM-A84M	-0.1033531	0.148102225	Low	0.148102
TCGA-TM-A84O	-0.1007719	0.139589498	High	-0.10077
TCGA-TM-A84Q	-0.2333301	0.117245738	Low	0.117246
TCGA-TM-A84R	-0.1196822	0.13392929	Low	0.133929
TCGA-TM-A84S	-0.1154697	0.128051717	Low	0.128052
TCGA-TM-A84T	-0.2093032	0.119653118	Low	0.119653
TCGA-TQ-A7RF	-0.2116886	0.12261796	Low	0.122618
TCGA-TQ-A7RG	-0.0966538	0.122576339	Low	0.122576
TCGA-TQ-A7RH	-0.2044714	0.112499154	Low	0.112499
TCGA-TQ-A7RI	-0.0912784	0.12291095	Low	0.122911
TCGA-TQ-A7RJ	-0.1964704	0.116593659	Low	0.116594
TCGA-TQ-A7RK	-0.2359517	0.131806308	Low	0.131806
TCGA-TQ-A7RM	-0.1899108	0.165299188	Low	0.165299
TCGA-TQ-A7RN	-0.1061833	0.1391136	Low	0.139114
TCGA-TQ-A7RO	-0.0750008	0.130068787	High	-0.075
TCGA-TQ-A7RP	-0.0635524	0.126805909	High	-0.06355
TCGA-TQ-A7RQ	-0.1191145	0.132162108	Low	0.132162
TCGA-TQ-A7RR	-0.2138075	0.114823671	Low	0.114824
TCGA-TQ-A7RV	-0.2031556	0.129789832	Low	0.12979
TCGA-TQ-A7RW	-0.176824	0.113974283	High	-0.17682
TCGA-TQ-A8XE	-0.201775	0.124114324	Low	0.124114
TCGA-VM-A8C8	-0.2041476	0.119886247	Low	0.119886
TCGA-VM-A8C9	-0.2353496	0.131158731	High	-0.23535
TCGA-VM-A8CA	-0.1646462	0.128510202	High	-0.16465
TCGA-VM-A8CB	-0.1692803	0.125333633	High	-0.16928
TCGA-VM-A8CD	-0.0685878	0.126566822	High	-0.06859
TCGA-VM-A8CE	-0.1230211	0.131301729	Low	0.131302
TCGA-VM-A8CF	-0.2393235	0.0936117	Low	0.093612
TCGA-VM-A8CH	-0.1791014	0.118159138	Low	0.118159
TCGA-VV-A829	-0.139893	0.133351561	Low	0.133352
TCGA-VV-A86M	-0.216572	0.116649717	High	-0.21657
TCGA-VW-A7QS	-0.0921536	0.146105688	Low	0.146106
TCGA-VW-A8FI	-0.1299575	0.135083845	High	-0.12996
TCGA-W9-A837	-0.10031	0.132288908	Low	0.132289
TCGA-WH-A86K	-0.2267912	0.124838128	Low	0.124838
TCGA-WY-A858	-0.1991575	0.113048684	High	-0.19916
TCGA-WY-A859	-0.2220669	0.135099968	Low	0.1351
TCGA-WY-A85A	-0.2049927	0.119928492	Low	0.119928
TCGA-WY-A85B	-0.2237222	0.113830291	Low	0.11383
TCGA-WY-A85C	-0.2215165	0.124668417	Low	0.124668
TCGA-WY-A85D	-0.2162587	0.111641091	Low	0.111641
TCGA-WY-A85E	-0.2217343	0.139796027	Low	0.139796
