# Supplementary figures and images for: Novel Cuproptosis-Related Gene Signature for Precise Identification of High-Risk Populations in Low-Grade Gliomas
Source: Mediators Inflamm. 2023 Feb 13;2023:6232620. doi: 10.1155/2023/6232620 (PMC9940981; doi:10.1155/2023/6232620)

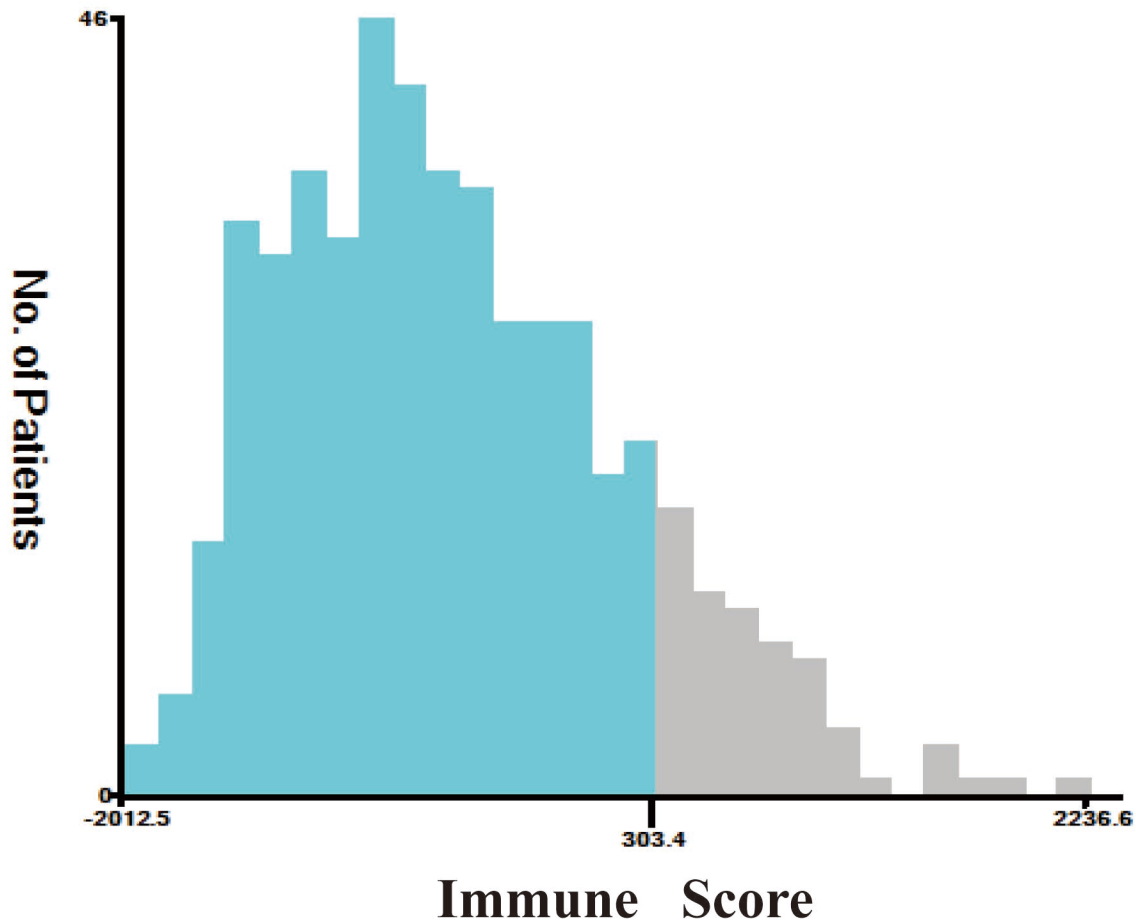

**Supplementary Figure 1. X-tile plots:** Cutoff values for immune scores

Supplement: Supplementary Materials — Supplementary Figure 1: X-tile plots: cutoff values for immune scores. Supplementary Material 2: TIDE score. [file 6232620.f1.zip › Supplementary-fig-1.pdf]
